# Expression of Cancer Testis Antigens in Colorectal Cancer: New Prognostic and Therapeutic Implications

**DOI:** 10.1155/2016/1987505

**Published:** 2016-08-18

**Authors:** Maciej Tarnowski, Michał Czerewaty, Anna Deskur, Krzysztof Safranow, Wojciech Marlicz, Elżbieta Urasińska, Mariusz Z. Ratajczak, Teresa Starzyńska

**Affiliations:** ^1^Department of Physiology, Pomeranian Medical University, 70-111 Szczecin, Poland; ^2^Department of Gastroenterology, Pomeranian Medical University, 70-111 Szczecin, Poland; ^3^Department of Biochemistry and Medical Chemistry, Pomeranian Medical University, 70-111 Szczecin, Poland; ^4^Department of Pathomorphology, Pomeranian Medical University, 70-111 Szczecin, Poland; ^5^Department of Regenerative Medicine, Warsaw Medical University, 02-091 Warsaw, Poland

## Abstract

*Background*. While cancer/testis antigens (CTAs) are restricted in postnatal tissues to testes and germ line-derived cells, their role in cancer development and the clinical significance of their expression still remain to be better defined.* Objective*. The aim of this study was to investigate the level of CTA expression in colon samples from patients with colorectal cancer (CRC) in relation to patient clinical status.* Methods*. Forty-five patients with newly diagnosed colorectal cancer were included in the study. We selected a panel of 18 CTAs that were previously detected in CRC as well as some new gene candidates, and their expression was detected at the mRNA level by employing RQ-PCR. Additionally, we evaluated CTA expression in three colon cancer cell lines (CL-188, HTB-39, and HTB-37) after exposure to the DNA methylation-modifying drug 5-azacytidine.* Results*. We report that 6 out of 18 (33%) CTAs tested (MAGEA3, OIP5, TTK, PLU1, DKKL1, and FBXO39) were significantly (*p* < 0.05) overexpressed in tumor tissue compared with healthy colon samples isolated from the same patients.* Conclusions*. Moreover, we found that MAGEA3, PLU-1, and DKKL expression positively correlated with disease progression, evaluated according to the Dukes staging system. Finally, 5-azacytidine exposure significantly upregulated expression of CTAs on CRC cells, which indicates that this demethylation agent could be employed therapeutically to enhance the immune response against tumor cells.

## 1. Introduction

In postnatal life, the cancer/testis antigens (CTAs) are typically restricted to the testes; however they may be aberrantly expressed in several types of cancers. There are more than 100 CTA genes reported to date, and their specific expression pattern makes them useful as markers of malignancies [1–5]. Although they seem to be involved in spermatogenesis and fertilization, the biological functions of several CTAs remain poorly understood. CTA expression varies and has different frequencies among different tumor types. For example, ovarian cancer, melanoma, and lung cancer are tumors with relatively frequent CTA expression, while, colon, renal, hematopoietic malignancies and pancreatic cancers are considered tumors with a low frequency of CTA expression [6, 7].

The abnormal expression of CTA on tumor cells is intriguing. On the one hand, it seems to support the so-called “embryonic rest hypothesis of cancer development.” Specifically, during the 19th and early 20th centuries, it was proposed that cancer originates in populations of cells that are left in a dormant state in developing organs during embryogenesis. This hypothesis was initially postulated by Recamier (1829), Remak (1854), and Virchow (1858) and later elaborated by Durante (1874) and Cohnheim (1875), who suggested that adult tissues contain embryonic remnants that normally lie dormant but that can be “activated” to become cancerous. In agreement with these theories, Wright (1910) proposed a germinal cell origin of Willms tumor (nephroblastoma), and Beard (1911) proposed that some tumors may arise from displaced trophoblasts or germ cells (reviewed in [8]).

On the other hand, abnormal expression of these CTAs in malignant cells could be a sign of reactivation of the silenced so-called “gametogenic program” [8, 9]. In cancer tissue, CTAs may play an important role in tumor development, proliferation, and antiapoptotic mechanisms [10]. While CTAs are mostly expressed in testes, which are considered immunologically privileged areas, their presence in cancer cells may elicit humoral and cell-mediated immune responses [11]. This is of potential clinical importance, since CTAs present on the surface of cancer cells could be potent targets for immune-based anticancer approaches.

Colorectal cancer (CRC) is the third most common cancer and the fourth most common cause of mortality worldwide, accounting for 9% of all malignancies [12, 13]. CRC results from the progressive accumulation of genetic and epigenetic alterations that lead to the transformation of normal colonic epithelium to colon adenocarcinoma. Both hereditary and environmental factors are involved in the development of CRC, and the majority of cases result from chromosomal instability involving the APC, K-ras, or p53 genes [14]. CRC usually occurs in later life, with an average age at diagnosis of around 70, and the vast majority of patients are diagnosed after the age of 50. When treated early by surgical or endoscopic resection, the disease has a good prognosis, but when it is present at a more advanced stage, the prognosis is generally poor [12, 13].

In the present study we evaluated the expression of a panel of CTAs, comprising 18 genes in three CRC cell lines and 45 patient samples. The selected panel of CTAs evaluated was previously detected in CRC; however, we also included new gene candidates. Here, we report that some CTAs are potential markers of CRC and that their expression correlates with disease progression. What is most important, CTAs are potential targets for immune cells, and their expression increased upon treatment with 5-azacytidine. In support of this notion, 5-azacytidine has been reported to upregulate CTA expression in certain cancer cell lines, which may render the cancer cells susceptible to the immune response [15].

## 2. Material and Methods

### 2.1. Patients

Forty-five patients with newly diagnosed colorectal cancer at the Department of Gastroenterology, Pomeranian Medical University in Szczecin, were included in the study. The median age of the patients was 75 years (range, 40–89; IQR, 17), and 58% of the patients were male. All patients had a malignancy confirmed by histology and gave written informed consent to participate in the study. The study conforms to the Code of Ethics of the World Medical Association (Declaration of Helsinki), printed in the British Medical Journal (18 July 1964). Twelve tumors (27%) were located in the rectum, 18 (44%) in the sigmoid colon, and 15 (33%) in the right colon. For statistical analysis, carcinomas were divided into rectal and nonrectal lesions. None of the patients received radiotherapy or immunotherapy before biopsy. Patients were also categorized according to smoking and body mass index (BMI). Clinical data covered localization of the tumor, lymph node involvement, the presence of distant metastases, and stage. Tissue samples from colon cancer and normal colonic mucosa, located 5 cm away from the tumor edge, were obtained at the time of colonoscopy. Tissue fragments were cut into small fragments and immediately frozen in RNAlater^®^ (Life Technologies, Inc., Grand Island, NY). Samples were stored at −80°C until the time of mRNA extraction. The stage and histological type of cancer were assessed from routine examination of paraffin sections obtained from surgical specimens. Evaluation of the stage was performed according to the criteria of Dukes [16]. There were 3 (7%) tumors in Dukes stage A, 16 (36%) in stage B, 11 (27%) in stage C, and 10 (22%) in stage D. The tumors were predominately moderately differentiated (G2; *n* = 36, 84%). Patient data are summarized in Table 1.

### 2.2. Cell Lines

We used the human colon cancer cell lines, CL-188 (LS 147T), HTB-39 (SK-CO-1), and HTB-37 (Caco-2), which were obtained from ATCC. Cells were cultured in Eagle's Minimum Essential Medium (EMEM) 1640 (Sigma-Aldrich, St. Louis, MO), supplemented with 100 IU/mL penicillin, 10 *μ*g/mL streptomycin, and 50 *μ*g/mL neomycin (Life Technologies) in the presence of 10% heat-inactivated fetal bovine serum (FBS, Life Technologies). The cells were cultured in a humidified atmosphere at 37°C in 5% CO_2_ at an initial cell density of 2.5 × 10^4^ cells/flask, and the medium was changed every 48 hours.

### 2.3. AzacytidineTreatment

Cells were treated with 5-azacytidine (Sigma-Aldrich) at a concentration of 5 *μ*M for 48 h. After the indicated time, the cells were lysed, and the RNA was isolated.

### 2.4. RNA Isolation

Tissues were stored in RNAlater Solution (Life Technologies) at −80°C. After defrosting, the tissues were rinsed with PBS buffer (Qiagen, Valencia, CA.) for 1 min to remove any residual RNAlater Solution. Samples were homogenized with the Ultra-Turrax T-10 basic dispersing tool in 600 mL RLT buffer (Qiagen, Valencia, CA.) for 5 min at 30,000 rpm/min. Total RNA was extracted from homogenates using the RNeasy Fibrous Tissue Mini Kit (Qiagen) in accordance with the manufacturer's protocol. The concentration and purity of RNA samples were determined by measuring the absorbance using a spectrophotometer (PerkinElmer Lambda Bio). In the next step, 0.3 *μ*g of RNA from each sample was reverse transcribed into cDNA in a total volume of 20 *μ*L with the RevertAid First Strand cDNA Synthesis Kit (Thermo Scientific, Waltham, MA) according to the manufacturer's instructions.

### 2.5. Real-Time Quantitative Reverse Transcription PCR (RQ-PCR)

Quantitative assessment of mRNA levels was performed by real-time RT-PCR on an ABI 7500 Fast instrument with Power SYBR Green PCR Master Mix reagent (Life Technologies). Real-time conditions were as follows: 95°C (15 sec), 40 cycles at 95°C (15 sec), and 60°C (1 min). According to melting point analysis, only one PCR product was amplified under these conditions. The relative quantity of a target, normalized to the endogenous control *β*-2 microglobulin and *β*-actin as internal calibrators, was calculated as the fold difference and further processed using statistical analysis.

### 2.6. Statistical Analysis

CTA expression data were compared between samples from colon cancer and normal colonic mucosa with the Wilcoxon signed-rank test. Data were analyzed as cancer tissue absolute expression (AE) and cancer tissue relative expression (RE) to normal tissue in the same patient, calculated as the ratio of expression levels: cancer tissue/normal tissue. The Mann-Whitney test was used to compare expression between groups of patients. Correlations between expression and other quantitative or rank variables (including Dukes stages) were analyzed using the Spearman rank correlation coefficient (*R*_*s*_). *p* < 0.05 was considered statistically significant.

## 3. Results

### 3.1. Expression of CTAs in Colon Cancer Cell Lines

**Figure 1:**
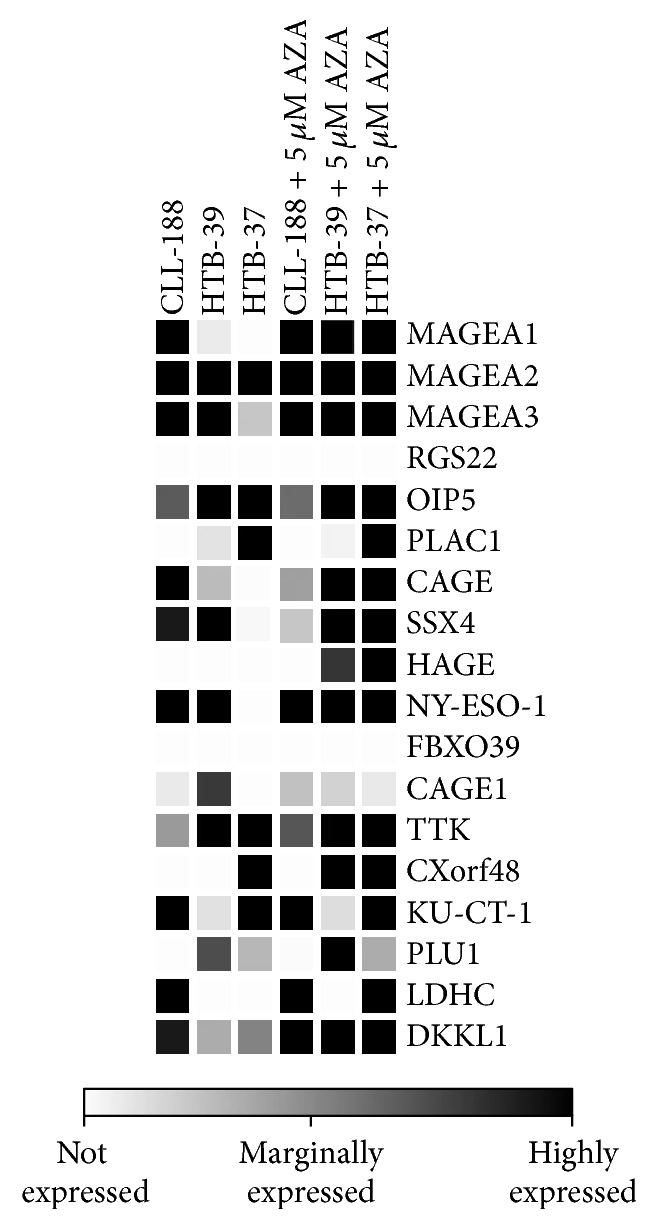
Real-time PCR (RQ-PCR) expression analysis of three colon cancer cell lines: CL-188, HTB-37, and HTB-39. A panel of 18 CTAs was tested, and their expression was compared with normal colon epithelium (left side). The same CTAs were evaluated after 5-azacytidine treatment at 5 *μ*M for 48 h (right side). The experiment was repeated three times with similar results.

We designed a panel of CTA candidates previously shown to be expressed in colorectal cancer (MAGEA1, A2, and A3; OIP5; PLAC1; CAGE; SSX4; HAGE; NY-ESO-1; and FBXO39) [17–20] and included some promising new targets (CAGE1, TTK, CXorf48, KU-CT-1, PLU1, LDHC, DKKL1, and RGS22), which are expressed in lung cancer, multiple myeloma, esophageal squamous cell carcinoma, breast cancer, melanoma, and head and neck squamous cell carcinoma [21–23]. We first tested expression of these CTAs in the three colon cancer adenocarcinoma cell lines CL-188, HTB-39, and HTB-37. This data is presented in the form of a heatmap in Figure 1. While HTB-37 and HTB-39 cell lines were of male patient origin, CL-188 was derived from a female patient. Moreover, CL-188 and HTB-37 were isolated from primary tumors, and HTB-39 was derived from a metastatic site in the peritoneum. CTA expression was measured as fold difference and compared with normal colon epithelium obtained during tumor biopsy. We noted that colon cancer cell lines do not uniformly express CTAs, and there is some degree of variability in the CTAs present among the three cell lines tested. RQ-PCR revealed that CL-188 is the highest CTA-expressing cell line, in which 9/18 (50%) of the CTAs evaluated in this study were overexpressed. By contrast, HTB-37 and HTB-39 overexpressed 6/18 (33%) and 7/18 (38%) of the CTAs, respectively.

### 3.2. Epigenetic Modification Leads to Increased Expression of CTAs in Colon Cancer Cell Lines

The expression of several CTAs is epigenetically controlled and regulated at the promoter level by the DNA methylation state. Thus, we exposed all the three cell lines to a methylation-modifying drug, 5-azacytidine (5-AzaC), and observed that several CTAs were expressed in response to a subtoxic dose (5 *μ*M) of this compound (Figure 1). We also observed that CRC cell lines did not respond equally to this DNA-demethylating drug. HTB-39 and HTB-37 cell lines increased the number of overexpressed CTAs from 7 to 11 and from 6 to 12, respectively, and expressed* de novo* several CT genes, including MAGEA1, CAGE, HAGE, and DKKL1. By contrast, except for DKKL1, the CL-188 cell line did not reactivate any of the CTAs evaluated in our study.

### 3.3. Expression of CTAs in Clinical Samples

Next, we evaluated a panel of 18 CTAs in 45 patients. From all patients we collected tissue from the tumor and samples from the normal adjacent colonic mucosa, which served as control. RNA was extracted from each individual sample, both normal and cancerous, and reverse transcribed. Subsequently, CTA expression was assessed by quantitative real-time PCR, and expression of *β*-actin together with *β*-microglobulin served as endogenous controls. When the Wilcoxon signed-rank test was used, we noted that 6 of 18 (33%) CTAs tested, MAGEA3, OIP5, TTK, PLU1, DKKL1, and FBXO39, were significantly (*p* < 0.05) overexpressed in the tumor compared with normal tissue located ~5 cm away from the primary lesion. At the same time, PLAC1 was overexpressed in tumors, but with borderline significance (*p* = 0.07) (Table 2).

**Figure 2:**
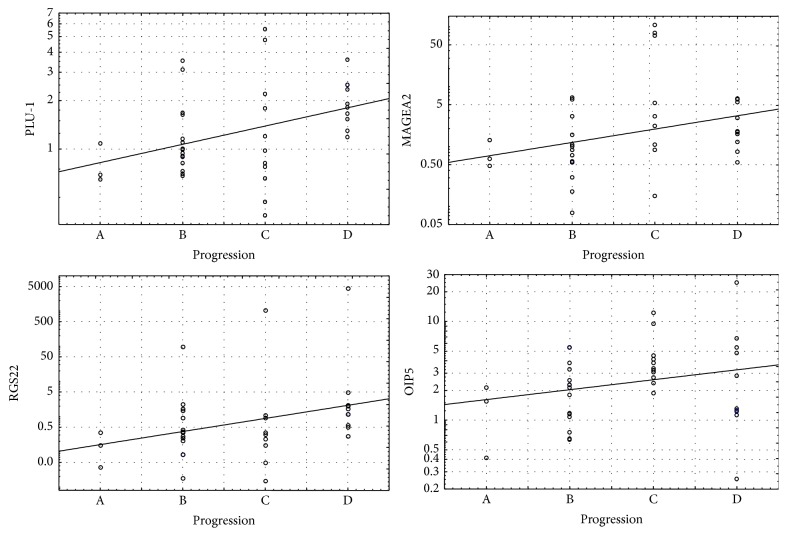
Scatterplots showing correlation between disease progression (according to Dukes staging system) and CTAs expression. Six out of 18 tested CTAs corresponded with advance of the disease, and four of them (PLU1, OIP5, RGS22, and MAGE-A2) showed positive correlation with relative expression (RE) with *p* value <0.05.

In further statistical analysis, we became interested in the association between CTA overexpression and cancer progression according to the Dukes staging system. A Spearman rank correlation test showed that absolute expression (AE) of most of the significantly overexpressed CTAs (DKKL1, PLAC1, and MAGEA3) correlated positively with disease progression (Table 3 and Figure 2). At the same time, PLU-1, MAGEA2, and RGS22 showed statistically significant positive correlations between the relative expression (RE) and disease progression (*p* < 0.05). Additionally, we found a borderline positive correlation between the RE for OIP5 (*p* = 0.086) and NY-ESO-1 (*p* = 0.051). However, the RE of OIP5 was significantly higher (*p* = 0.03) in advanced stages of the disease (stages C or D) compared with stages A or B (Table 4). By contrast, TTK and FBXO39, which were overexpressed in cancer tissue, did not show any association with disease progression.

Table 5 shows a significant association between clinical characteristics and CTA expression. Of note, a significantly higher RE for PLU1, KU-CT-1, and RGS22 antigens in cancer tissue was observed in patients with distant metastases. When AE was taken into account, overexpression of PLU1, KU-CT-1, DKKL1, SSX4, and PLAC1 also demonstrated statistical significance.

**Figure 3:**
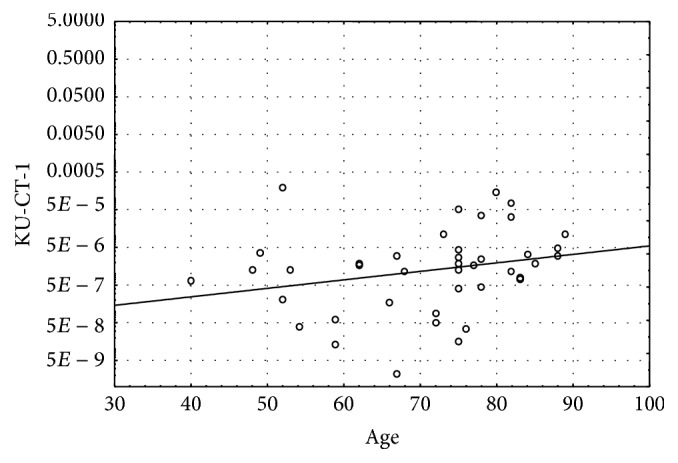
Positive correlation between absolute expressions (AE) of KU-CT-1 in cancer tissue and patients' age (*R*_*s*_ = 0.43, *p* = 0.003).

Additional analysis showed a correlation between KU-CT-1 expression in cancer tissue and patient age. Specifically, its expression in cancer tissue (AE) progressively increased with age (*R*_*s*_ = 0.43, *p* = 0.003, Figure 3). What is also interesting, we noted that the AE of three CTAs were associated with gender: MAGE A1, CAGE, and NY-ESO-1 were significantly overexpressed in cancer tissue of female patients. Moreover, two tested CTAs were significantly downregulated in smoking patients (MAGEA1, AE; FBXO39, AE and RE) and one CTA (LDHC) seemed to be upregulated (AE) in patients with obesity.

## 4. Discussion 

The salient observation of our study is the identification of new potential prognostic markers for CRC and the observation that 5-azacytidine enhances expression of CTAs in CRC cells. Thus, these observations are of both diagnostic as well as of potential therapeutic value.

CRC is the fourth most common carcinoma, with a high rate of mortality worldwide, and is the third highest cause of cancer-related death [13]. Over the last few decades, several studies focusing on cancer molecular markers have been performed; however, only a few such markers have attracted clinical interest. The obvious lack of clinical prognostic markers clearly reflects the lack of prognostic studies.

Nevertheless, certain CTAs have recently been reported to be significantly upregulated in CRC cells [19, 20]. Although the function of CTA genes is still largely unknown, partly due to their presence in multiple tumor types, their limited expression in normal tissue has made them putative molecular markers for cancer prognosis, diagnosis [5–7], and immunotherapy [10].

We selected a panel of 18 CTAs and first evaluated their expression in three CRC cell lines and observed that they showed relatively rare CTA expression (38–50%). Our results are in accordance with previous studies, where colon cancer cell lines also did not express CTAs frequently and uniformly [17]. Antigens overexpressed in colon cancer cell lines only partially paralleled the results obtained from primary tumor samples, and this lack of uniformity was also seen in our clinical samples. Nevertheless, MAGEA3, OIP5, and TTK were all highly expressed in CRC cell lines and at the same time significantly overexpressed in primary tumors (Table 2).

In our study we noted statistically significant expression of some CTAs in CRC samples; however, none were expressed in all cancer samples. Nevertheless, our study shows that there are some CTAs expressed at higher frequencies (*p* < 0.05) in CRC patient samples (MAGEA3, OIP5, TTK, PLU1, DKKL1, and FBXO39, Table 2). Interestingly, when the Spearman rank correlation test was employed, we found that, except for FBXO39 and TTK, overexpression of these genes corresponded with more advanced disease (Table 3). Additionally, MAGEA2 expression also corresponded with disease progression, which makes the MAGE family of antigens the most frequently overexpressed in CRC. MAGEA3 is generally expressed in a variety of solid tumors [24, 25] and its presence was detected in 20% of CRCs. Here, we confirmed its high expression, both in our CRC cell lines and in patient samples. However, as reported in the past [26], we also did not find a correlation between MAGEA3 expression and age, sex, or histological type of cancer. Li et al. and Shantha Kumara et al. showed expression of MAGEA3 in 27.3% and 28% of CRC patients, respectively [19, 20], and MAGEA3 was also detected in 13% of CRC samples by Alves et al. [27] and postulated to elicit an immunological response.

Interestingly, besides MAGE-A3, Shantha Kumara et al. found increased expression of PLAC1 in the majority (83%) of colon cancer cell lines and in 12.8% of patient samples [20]. In our hands, MAGEA3 and PLAC1 were not only overexpressed in CRC, but what is even more important, their expression increased together with disease progression, according to the Dukes classification. Thus, MAGEA3 and PLAC1 may have some prognostic value, as observed in nonsmall lung cancer [28], renal carcinoma [29], or prostate adenocarcinoma [30].

OIP5 was also significantly overexpressed in CRC cell lines and patient samples [31]. Reportedly, OIP5 also has some prognostic value, and its expression paralleled a poor prognosis in nonsmall cell lung cancer and esophageal cancer [32]. What is most important and a new observation from our studies, a similar correlation was found between expression of OIP5 in CRC and advanced stages of this cancer.

Furthermore, an interesting CTA overexpressed in our study is PLU1 (CT31), which belongs to the JARID1 histone demethylase family and plays an important role in histone methylation [33]. Ohta et al. showed that suppression of PLU1 may become a potential target for CRC treatment [34]. In our work PLU1 overexpression correlated with CRC progression. We also observed that it was expressed at higher levels in patients with distant metastases.

We also report here the high expression of TTK, both in CRC cell lines and in patient samples. TTK was previously reported to be highly expressed in prostate cancer [35] and Hodgkin's lymphoma [36]. Interestingly, mutations in the TTK gene were observed in CRCs with microsatellite instability [37], and its role is closely related to pancreatic cancer cell proliferation and malignant transformation [38].

Another CTA, DKKL1 (also known as SGY-1 (soggy-1)), is a protein related to the Dickkopf protein family that negatively regulates Wnt-mediated effects [39]. While DKKL1 has been shown to be expressed in melanoma and lung cancer, surprisingly it was not detected in 20 cDNA library samples from CRC patients [40]. In our study DKKL1 was significantly overexpressed in cancer tissue and correlated positively with disease stage.

There is a growing body of evidence that cancer, including CRC, may be considered as a stem cell disease that results from the existence of self-renewing and pluripotent cancer-initiating cells. Among the cell populations identified, very small embryonic-like stem cells residing in adult tissues are potential candidates for such malignant transformation [8]. Several CTAs are expressed in human embryonic stem cells [8] and in very small embryonic-like stem cells isolated from adult tissues [41]. Yamada at al. showed in direct studies that cancer stem/initiating cells express a variety of CTAs. Among them, characteristic CTAs were detected in a side population of the colon cancer cell line SW480 [42]. In general, this suggests that CTAs would be potent targets for immunotherapy to destroy cancer stem cells.

Although colon cancer is regarded as a low-CTA-expressing tumor, there are several strategies by which the number of expressed CTAs may be increased. One method is the use of epigenetic drugs, specifically demethylating agents such as 5-azacytidine [15]. Amplification of CTA expression may in turn improve tumor recognition by the immune system. 5-Azacytidine is a potent DNA methyltransferase 1 inhibitor that, after being incorporated into DNA, produces global hypomethylation. In this paper, we demonstrated that 5-azacytidine treatment significantly upregulates CTA expression. Thus, our results are in line with previous studies in which several CTAs reactivated their expression upon treatment with methylation-modifying drugs [43]. This observation is of clinical relevance, as 5-azacytidine could be employed to increase the immune response against CRC.

In conclusion, several CTAs, such as MAGEA3, PLU-1, or DKKL, may serve as biomarkers of CRC, and since their expression correlates with advanced stages of the disease, they are of prognostic relevance. Finally, since 5-azacytidine treatment upregulates several CTAs on CRC cells, application of this DNA demethylating agent should be explored to enhance the immune response against CRC.

## Figures and Tables

**Table 1:** Clinical data of patients enrolled in the study.

Risk factors	
Age	Mean = 70 (range 40–88); median = 75; SD = 12.7; IQR = 17

Gender	Male (*n* = 26)
	Female (*n* = 19)

BMI	<30 (*n* = 26)
	>30 (*n* = 7)
	Unknown (*n* = 12)

Smoking	Yes (*n* = 17)
	No (*n* = 17)
	Unknown (*n* = 11)

Staging	A (*n* = 3)
	B (*n* = 16)
	C (*n* = 11)
	D (*n* = 10)
	Unknown (*n* = 5)

Localization	Colon, right (*n* = 15)
	Colon, left (*n* = 18)
	Rectum (*n* = 12)

Lymph node involvement	Yes (*n* = 13)
	No (*n* = 17)
	Unknown (*n* = 15)

Distant metastasis	Yes (*n* = 9)
	No (*n* = 27)
	Unknown (*n* = 9)

Surgical resection	Yes (*n* = 35)
	No (*n* = 3)
	Unknown (*n* = 7)

Grading	G1 (*n* = 3)
	G2 (*n* = 30)
	G3 (*n* = 2)
	Unknown (*n* = 10)

Total	45

**Table 2:** Comparison between CTA expressions in normal and cancer tissue obtained during colonoscopy.

	CTA	Median	IQR	*p* value
Cancer versus normal	MAGEA1	1.004268	3.557637	0.923
	MAGEA2	1.068458	2.669421	0.586
	**MAGEA3**	1.488304	1.660176	**0.003**
	**OIP5**	2.520143	2.942988	**p** < 0.001
	SSX4	1.70662	4.9859	0.365
	PLAC1	1.467123	2.930341	0.072
	CAGE1	1.183878	2.159953	0.188
	**TTK**	3.512544	4.599179	**p** < 0.001
	CXorf48	1.000834	6.828382	0.538
	KU-CT-1	2.57151	16.58306	0.128
	HAGE	1.139154	3.847637	0.177
	**PLU1**	1.178954	0.914003	**0.018**
	LDHC	1.559463	8.283443	0.232
	NY-ESO-1	1.443452	4.199511	0.604
	**DKKL**	1.365828	2.845266	**0.040**
	**FBXO39**	2.136684	1.850426	**p** < 0.001
	RGS22	0.485704	1.391908	0.251
	CAGE	1.041833	2.008881	0.879

RNA was isolated from cancer tissue and normal tissue located ~5 cm away and reverse transcribed and CTA expression was analyzed by real-time PCR. Differences with *p* value <0.05 (Wilcoxon signed-rank test) are in bold font.

**Table 3:** Analysis of CTA expression in relation to disease progression (Dukes staging in CRC).

Variable	CTA	RE		AE	
		*R*_*s*_ value	*p* value	*R*_*s*_ value	*p* value
Staging	MAGEA1	0.14	0.417	0.04	0.800
	**MAGEA2**	0.40	**0.017**	0.13	0.408
	**MAGEA3**	0.15	0.254	0.33	**0.039**
	OIP5	0.28	0.086	0.01	0.957
	SSX4	0.18	0.276	0.16	0.336
	**PLAC1**	0.19	0.240	0.34	**0.034**
	CAGE1	0.19	0.235	0.25	0.122
	TTK	0.05	0.750	0.25	0.116
	CXorf48	0.28	0.082	0.30	0.080
	KU-CT-1	0.26	0.102	0.22	0.195
	HAGE	−0.14	0.402	−0.14	0.373
	**PLU-1**	0.46	**0.003**	0.07	0.680
	LDHC	−0.21	0.195	0.32	0.071
	NY-ESO-1	0.31	0.051	0.05	0.777
	**DKKL**	0.14	0.391	0.33	**0.037**
	FBXO39	−0.04	0.818	0.04	0.812
	**RGS22**	0.40	**0.015**	0.23	0.145
	CAGE	0.08	0.635	0.21	0.200

The Spearman rank correlation coefficient was used to analyze the correlation between the stage and CTA expression level in cancer tissue. Both relative expression (RE) and absolute expression (AE) were calculated. Correlations with *p* value <0.05 are in bold font.

**Table 4:** Analysis of CTA expression in relation to disease progression.

Variable	CTA	Median CD	Median AB	RE *p* value
Staging C + D versus A + B	MAGEA1	1.183	0.820	0.188
	**MAGEA2**	2.220	0.706	**0.009**
	MAGEA3	1.881	1.488	0.085
	**OIP5**	3.168	2.114	**0.034**
	SSX4	2.313	0.959	0.350
	*PLAC1*	2.702	1.050	0.336
	CAGE1	2.498	1.034	0.144
	*TTK*	4.756	3.378	0.101
	CXorf48	1.817	0.797	0.058
	KU-CT-1	3.569	1.633	0.605
	HAGE	1.139	0.929	0.560
	**PLU-1**	1.659	0.939	**0.044**
	LDHC	2.004	0.794	0.157
	NY-ESO-1	2.946	0.827	0.129
	*DKKL*	1.844	1.184	0.382
	*FBXO39*	2.204	1.877	0.517
	RGS22	0.877	0.353	0.159
	CAGE	1.305	0.703	0.424

Mann-Whitney *U* test was applied to compare late stages C + D with stages A and B. Correlations with *p* value <0.05 are in bold font.

**Table 5:** Analysis of CTA expression in relation to clinicopathological features.

CTA	Clinical feature		AE			RE		
			IQR	Median	*p* value	IQR	Median	*p* value
KU-CT-1	Distant metastases	−	1.90*E* − 06	7.90*E* − 07	**0.025**	3.80*E* + 00	1.10*E* + 00	**0.030**
		+	1.70*E* − 06	2.80*E* − 06		4.60*E* + 01	1.20*E* + 01	
PLU1		−	1.10*E* − 02	9.60*E* − 03	0.060	9.10*E* − 01	9.80*E* − 01	**0.002**
		+	1.30*E* − 02	1.90*E* − 02		8.30*E* − 01	1.90*E* + 00	
DKKL		−	1.80*E* − 05	1.30*E* − 05	**0.029**	1.70*E* + 00	1.20*E* + 00	0.521
		+	4.10*E* − 04	9.30*E* − 05		3.30*E* + 00	1.00*E* + 00	
RGS22		−	1.50*E* − 05	4.90*E* − 06	**0.046**	7.90*E* − 01	2.80*E* − 01	**0.005**
		+	2.20*E* − 05	2.10*E* − 05		1.50*E* + 00	1.60*E* + 00	
SSX4		−	5.50*E* − 06	6.50*E* − 07	**0.039**	6.90*E* + 00	8.20*E* − 01	0.234
		+	2.00*E* − 06	2.00*E* − 06		2.60*E* + 00	2.60*E* + 00	
PLAC1		−	6.40*E* − 05	3.20*E* − 05	**0.014**	2.30*E* + 00	1.10*E* + 00	0.281
		+	1.60*E* − 04	1.20*E* − 04		3.20*E* + 00	2.80*E* + 00	

MAGEA1	Gender	−	4.00*E* − 05	1.90*E* − 05	**0.031**	6.20*E* + 00	1.50*E* + 00	0.574
		+	1.20*E* − 05	8.00*E* − 06		8.20*E* − 01	9.20*E* − 01	
CAGE		−	5.60*E* − 05	2.80*E* − 05	**0.039**	5.20*E* + 00	1.70*E* + 00	0.141
		+	5.50*E* − 05	1.10*E* − 05		1.30*E* + 00	7.70*E* − 01	
NY-ESO-1		−	1.50*E* − 06	2.70*E* − 06	**0.046**	3.10*E* + 00	2.50*E* + 00	0.078
		+	7.70*E* − 07	7.70*E* − 07		1.30*E* + 00	1.30*E* + 00	

MAGEA1	Smoking	−	3.90*E* − 05	1.90*E* − 05	**0.034**	3.80*E* + 00	1.20*E* + 00	0.375
		+	1.20*E* − 05	6.50*E* − 06		1.00*E* + 00	9.70*E* − 01	
FBXO39		−	2.50*E* − 04	1.60*E* − 04	0.020	1.60*E* + 00	2.60*E* + 00	**0.009**
		+	1.40*E* − 04	5.80*E* − 05		1.30*E* + 00	1.30*E* + 00	

LDHC	Obesity BMI > 30	−	5.80*E* − 06	2.00*E* − 06	**0.018**	1.40*E* + 01	1.80*E* + 00	1.000
		+	8.70*E* − 05	6.70*E* − 05		7.70*E* + 00	2.30*E* + 00	

Cxorf48	Lymph node involvement	−	1.90*E* − 06	5.20*E* − 07	**0.116**	4.20*E* + 00	6.80*E* − 01	**0.034**
		+	5.10*E* − 06	2.80*E* − 06		8.40*E* + 01	7.30*E* + 00	

Differences with *p* value <0.05 are in bold font.
